# ﻿Morphological and phylogenetic analyses reveal new species and records of *Fusarium* (Nectriaceae, Hypocreales) from China

**DOI:** 10.3897/mycokeys.116.150363

**Published:** 2025-04-07

**Authors:** Congcong Ai, Qiyun Liu, Yaling Wang, Zhaoxue Zhang, Duhua Li, Yun Geng, Xiuguo Zhang, Jiwen Xia

**Affiliations:** 1 Shandong Provincial Key Laboratory for Biology of Vegetable Diseases and Insect Pests, College of Plant Protection, Shandong Agricultural University, Taian, 271018, China Shandong Agricultural University Taian China; 2 Institute of Crop Germplasm Resources, Shandong Academy of Agricultural Sciences, Jinan, 250100, China Institute of Crop Germplasm Resources, Shandong Academy of Agricultural Sciences Jinan China; 3 College of Agriculture and Forestry, Linyi University, Linyi, Shandong, 276000, China Linyi University Linyi China

**Keywords:** *Fusariumincarnatum*-*equiseti* species complex, multigene phylogeny, new taxa

## Abstract

Species of *Fusarium* are important phytopathogens, saprobes, and endophytes around the world. Some species can affect plant health and cause yield loss of economic plants. *Fusarium* species are widely distributed in China, and many species were found from different plant hosts. The *Fusariumincarnatum*-*equiseti* species complex (FIESC) is one of the most significant species complexes within the genus. Based on morphological and three-gene (*cal*, *rpb2*, and *tef1*) phylogenetic analyses, two new species are in the Incarnatum clade, and two new host records are identified and described, viz. *Fusariumfici***sp. nov.**, *Fusariumxylosmatis***sp. nov.**, *Fusariumfecundum*, and *Fusariumweifangense*.

## ﻿Introduction

Johann Heinrich Friedrich Link first proposed the genus *Fusarium* (Nectriaceae, Hypocreales) in 1809 and typified it with *Fusariumroseum* (= *F.sambucinum*), with falcate or banana-shaped macroconidia and oval, subglobose, or kidney-shaped microconidia (Link 1809; Gams et al. 1997; Leslie and Summerell 2006; Liu et al. 2023; Zhang et al. 2023a). *Fusarium* is one of the most renowned and extensively spread genera in the Kingdom Fungi because of its morphological and phylogenetic diversity (Leslie and Summerell 2006; Sandoval-Denis et al. 2018; Crous et al. 2021). *Fusarium* species are known as plant pathogens, endophytes, and saprophytes (Leslie et al. 1990; Bacon and Yates 2006; Maryani et al. 2019; He et al. 2024). More than 1800 epithets of *Fusarium* have been listed in Index Fungorum (https://www.indexfungorum.org), but many species of *Fusarium* were identified solely based on morphological studies. Excessive overlap of conidial characteristics makes it difficult to morphologically distinguish *Fusarium* species. Currently, *Fusarium* taxonomy is dominated by morphological and molecular phylogenetic studies (Crous et al. 2021; He et al. 2024).

At present, *Fusarium* contains 23 monophyletic species complexes and several single-species lineages (Xia et al. 2019; O’Donnell et al. 2020; Geiser et al. 2021; He et al. 2024). The FIESC includes over 30 recognized phylogenetic species (O’Donnell et al. 2009; Villani et al. 2016; Maryani et al. 2019; Santos et al. 2019; Wang et al. 2019; Xia et al. 2019). Based on the haplotype nomenclature system, O’Donnell et al. (2009) implemented an informal classification system for FIESC and introduced the Equiseti and Incarnatum clades. The *Fusariumcamptoceras* species complex (FCAMSC) was proposed for three lineages that are sister clades to the FIESC by phylogenetic studies by Xia et al. (2019). However, Han et al. (2023) included the FCAMSC in FIESC as the Camptoceras clade because the FCAMSC and FIESC clearly represent a distinct evolutionary lineage that is strongly supported by the phylogenomic tree. Thus, FIESC comprises three clades, viz. Camptoceras, Equiseti, and Incarnatum clades.

In this study, samples were collected from Hainan, Sichuan, and Yunnan Provinces of China. Two new species and two new host records were identified and classified by multi-locus analysis of calmodulin (*cal*), RNA polymerase II second largest subunit (*rpb2*), and translations elongation factor 1-alpha (*tef1*) datasets. They were described and discussed based on their morphological characteristics along with their molecular sequence data.

## ﻿Materials and methods

### ﻿Strain isolation and preservation

Plant specimens with necrotic spots were collected from three provinces (Hainan, Sichuan, and Yunnan) of China in 2023. Pure colonies were obtained by tissue isolation techniques (Zhang et al. 2024). Fragments (25 mm^2^) were cut from the edges of diseased tissues, immersed in a 75% ethanol solution for 1 min, then rinsed in sterile water for 30 s and 10% sodium hypochlorite solution for 1 min. Fragments were rinsed three times with sterile water for 30 s, then using sterilized filter paper to absorb dry, placed on PDA for incubation at 25 °C for 3 days. The strains were preserved in 10% sterilized glycerol and stored them at 4 °C for future detailed studies. Specimens were deposited in the Herbarium of the Department of Plant Pathology, Shandong Agricultural University, Taian, China (HSAUP), and the Herbarium Mycologicum Academiae Sinicae, Institute of Microbiology, Chinese Academy of Sciences, Beijing, China (HMAS). The living ex-type cultures were deposited in the Shandong Agricultural University Culture Collection (SAUCC) and the China General Microbiological Culture Collection Center (CGMCC).

### ﻿DNA extraction, amplification, and sequencing

Total genomic DNA was extracted from fresh fungal mycelia grown on potato dextrose agar (PDA) after 7 days using the genomic DNA purification kit (OGPLF-400, GeneOnBio Corporation, Changchun, China) according to the product manual. The calmodulin (*cal*), RNA polymerase second largest subunit (*rpb2*), and translation elongation factor 1-alpha (*tef1*) gene loci were amplified using the primer pairs listed in Table 1 (Xia et al. 2019; Han et al. 2023). The reaction was performed in a 25 μL reaction volume, consisting of 12.5 μL of 2 × Hieff Canace® Plus PCR Master Mix (Cat. No. 10154ES03, Yeasen Biotechnology, Shanghai, China), 1 μL each of forward and reverse primer (TsingKe, Qingdao, China), and 1 μL of template genomic DNA, and at last replenished the total volume to 25 µL with double distilled water. PCR products were separated and purified using 1% agarose gel and Safe Red (RM02852 and RM19009 ABclonal, Wuhan, China) and UV light to visualize the fragments. Gel was extracted using a gel extraction kit (Cat. No. AE0101-C, Shandong Sparkjade Biotechnology Co., Ltd., Jinan, China) (Wang et al. 2023). The purified PCR products were sequenced by Youkang Company Limited (Zhejiang, China). All sequences generated in this study were deposited in GenBank under the accession numbers provided in Suppl. material 1.

**Table 1.** Molecular markers and their PCR primers and programs used in this study.

Loci	PCR Primers	Sequence (5′→3′)	PCR Cycles	References
*cal*	CL1	GARTWCAAGGAGGCCTTCTC	(94 °C: 30 s, 55 °C: 30 s, 72 °C: 15 s) × 35 cycles	O’Donnell et al. (2020)
	CL2A	TTTTTGCATCATGAGTTGGAC		
*rpb2*	5f2	GGGGWGAYCAGAAGAAGGC	(94 °C: 45 s, 57 °C: 45 s, 72 °C: 15 s) × 35 cycles	Liu et al. (1999)
	7cr	CCCATRGCTTGYTTRCCCAT		
*tef1*	EF-1	ATGGGTAAGGARGACAAGAC	(94 °C: 45 s, 55 °C: 45 s, 72 °C: 15 s) × 35 cycles	O’Donnell et al. (1998)
	EF-2	GGARGTACCAGTSATCATG		

### ﻿Phylogenetic analyses

The reference sequences were downloaded from NCBI’s GenBank. All sequences were initially aligned with the MAFFT v. 7 (http://mafft.cbrc.jp/alignment/server/) online service and MEGA 7.0. The concatenated aligned *cal*, *rpb2*, and *tef1* sequences were used for maximum likelihood (ML) and Bayesian inference (BI), which were run on RaxML-HPC2 with XSEDE v. 8.2.12 and MrBayes v. 3.2.7a with 64 threads on Linux (Zhang et al. 2024). For ML analyses, 100 rapid bootstrap replicates and the GTR+FO+I+G4m model as default parameters were used. For BI analyses, a fast bootstrap algorithm with an automatic stop option was performed (Zhang et al. 2023b). The SYM+G model for *cal*, the SYM+I+G model for *rpb2*, and the GTR+I+G model for *tef1* were selected and incorporated into the analyses. The Markov chain Monte Carlo (MCMC) analysis of the sequence data was performed over 5,000,000 generations, yielding 34,652 trees. Following the discard of 8,663 trees during the burn-in phase, the remaining trees were used to calculate posterior probabilities in the consensus trees.

### ﻿Morphological characterization

All isolates were inoculated on potato dextrose agar (PDA) medium and oatmeal agar (OA) medium. Colony morphology, pigmentation, and growth rates were recorded. The above and reverse of the PDA and OA flat plates were captured with the Alpha 6400L digital camera (Canon Powershot G7X, Canon, Tokyo, Japan) on the 7^th^ day. Used Carnation leaf agar (CLA; Fisher et al. 1982) medium to describe morphological features, such as shape, size, and septum number of the conidia (Wang et al. 2019). Used a stereomicroscope (Olympus SZ61, Olympus Corporation, Tokyo, Japan) and a microscope (Olympus BX53, Olympus Corporation, Tokyo, Japan) with Differential Interference Contrast (DIC) to observe the microscopic morphology. Stereomicroscope and microscope were equipped with BioHD-A20c color digital cameras (FluoCa Scientific, Shanghai, China) to capture the microscopic fungal structures. Microstructures were randomly measured using Digimizer software v5.6.0 (https://www.digimizer.com, accessed on 18 November 2024) and calculated the mean size (av.). The “n” represents the number of measurements.

## ﻿Results

### ﻿Phylogenetic analyses

**Figure 1.**
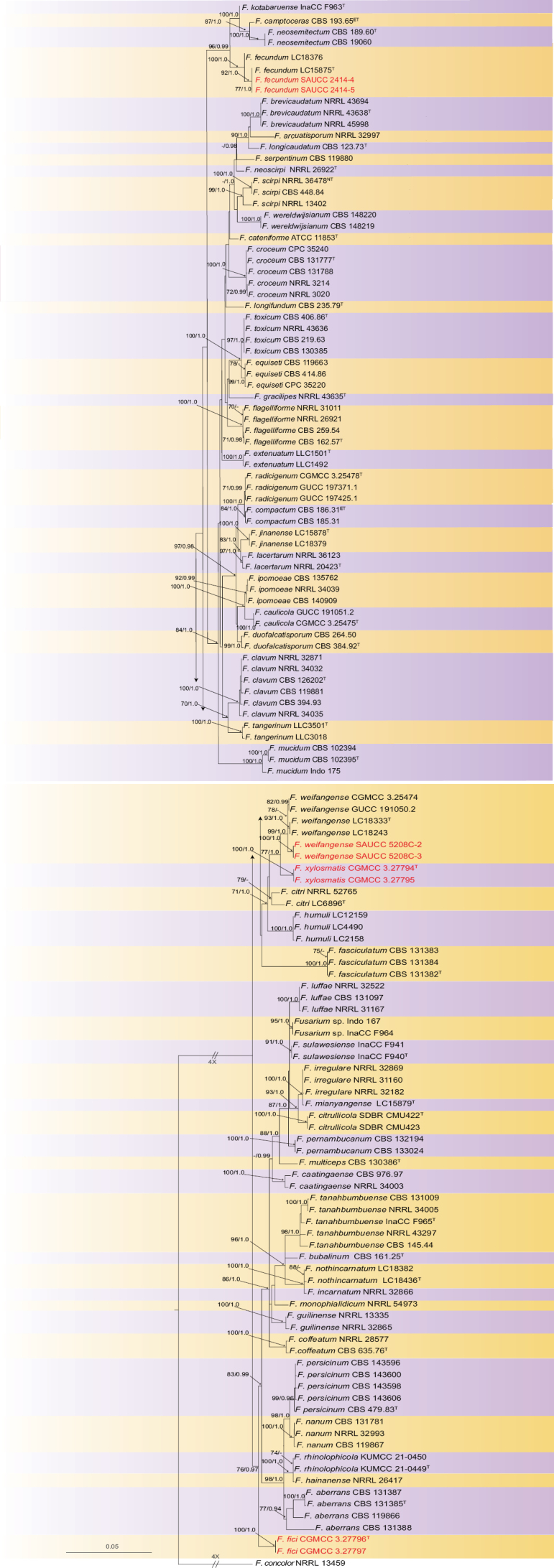
Phylogeny inferred based on the combined *cal*-*rpb2*-*tef1* sequence dataset of the *Fusariumincarnatum-equiseti* species complex (FIESC), with *Fusariumconcolor* (NRRL 13459) as the outgroup. The RAxML Bootstrap support values (MLBS ≥ 70%) and Bayesian posterior probabilities (BIPP ≥ 0.90) were shown at the nodes. Ex-type, ex-epitype, and ex-neotype strains were indicated by T, ET, and NT, respectively. Strains isolated in this study were indicated in red.

The combined dataset comprised 133 ingroup strains with *Fusariumconcolor* (NRRL 13459) as the outgroup. The final alignment comprised 1,654 concatenated characters, spanning from positions 1 to 535 (*cal*), 536 to 1,192 (*rpb2*), and 1,193 to 1,654 (*tef1*). The ML was carried out to be -9,907.383240. MrModelTest recommended using Dirichlet base frequencies for the *cal*, *rpb2*, and *tef1* data partitions. The alignment showed a total of 563 unique site patterns (*cal*: 156, *rpb2*: 168, *tef1*: 239). Based on the three-gene (*cal*, *rpb2*, and *tef1*) phylogeny, the 134 strains were classified into 57 species. The topology of the ML tree confirmed the topology obtained from BI, with only the ML tree presented (Fig. 1). Furthermore, single gene trees were evaluated, respectively, for FIESC (Suppl. material 2).

### ﻿Taxonomy

#### ﻿ Fusarium fecundum

Taxon classificationFungiHypocrealesNectriaceae

S.L. Han, M.M. Wang & L. Cai, Studies in Mycology 104: 87–148. 2023.

48320A27-F99C-5410-9B6D-4F082009DF44

Fig. 2

##### Description.

On CLA, conidiophores arising from aerial mycelia, 13–71 μm long, unbranched or irregularly branched, bearing terminal or lateral phialides, often reduced to single phialides; Periclinal thickening inconspicuous; Aerial conidia hyaline, smooth, rarely ovoid to falcate, on the apical half, the dorsal side is more curved than the ventral side, and the apical cell is either blunt or hooked, basal cell barely to distinctly notched. 1-septate conidia: (16–)22–21(–27) × 4–6 μm (av. 20 × 5 μm, n = 9); 2-septate conidia: (18–)21–28(–33) × 5–7 μm (av. 26 × 6 μm, n = 9); 3-septate conidia: (32–)33–36(–41) × 5–8 μm (av. 35 × 7 μm, n = 16); 4-septate conidia: (32–)37–43(–43) × 6–9 μm (av. 39 × 7 μm, n = 18); 5-septate conidia: (41–)43–48(–53) × 7–9 μm (av. 46 × 8 μm, n = 12).

**Figure 2.**
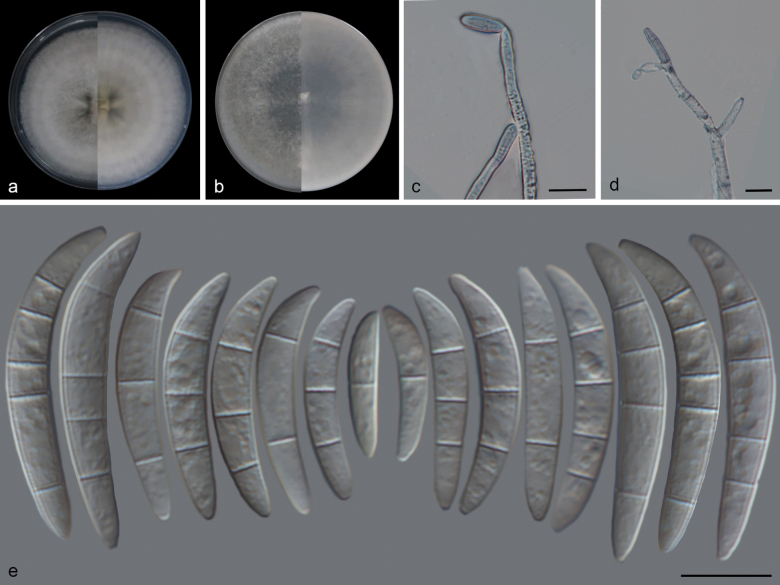
*Fusariumfecundum* (SAUCC 2414-4) **a** colony on PDA after 7 days at 25 °C (left: above, right: reverse) **b** colony on OA after 7 days at 25 °C (left: above, right: reverse) **c, d** conidiophore on aerial mycelium with monophialides **e** aerial conidia. Scale bars: 10 μm (**c–e**).

##### Culture characteristics.

Colonies on PDA incubated at 25 °C in the dark, reaching 84–90 mm diameter in 7 d; aerial mycelia dense, white, radiate, colony margin erose; reverse surface greyish yellow in the center, odor absent. On OA in the dark, occupying an entire 90 mm diameter in 7 d; surface white and aerial mycelia scant, crateriform, reverse white, odor absent.

##### Materials examined.

China • Yunan Province, Nanuo Mountain, on leaves of *Setariapalmifolia*, 3 March 2023, Q.Y. Liu (HSAUP41424, HSAUP51424), living cultures CGMCC 3.27792 = SAUCC 2414-4, CGMCC 3.27793 = SAUCC 2414-5.

##### Notes.

Phylogenetic analysis showed that isolates (SAUCC 2414-4 and SAUCC 2414-5) were closely related to *Fusariumfecundum* (LC15875, ex-type strain) (Fig. 1). There are no nucleotide position differences between *Fusariumfecundum* (SAUCC 2414-4) and *Fusariumfecundum* (LC15875, ex-type strain). Morphologically, *Fusariumfecundum* (SAUCC 2414-4) and *Fusariumfecundum* (LC15875, ex-type strain) are the lack of sporodochia. The aerial conidia of *Fusariumfecundum* (SAUCC 2414-4) are smaller than those of *Fusariumfecundum* (LC15875, ex-type strain). *Fusariumfecundum* was previously isolated from wheat and rice, and it has now been reported for the first time on *Setariapalmifolia* (Han et al. 2023).

#### ﻿ Fusarium fici

Taxon classificationFungiHypocrealesNectriaceae

Q.Y. Liu, X.G. Zhang & J.W. Xia sp. nov.

9C11FCAF-340D-555E-AD4D-45239F3E1CE6

MycoBank No: 856644

Fig. 3

##### Etymology.

Referring to the genus name of the host plant *Ficusfistulosa*.

##### Typus.

China • Hainan Province, Baoting Li and Miao Autonomous County, on leaves of *Ficusfistulosa*, 10 April 2023, Q.Y. Liu (HMAS 353395, holotype), ex-holotype culture CGMCC 3.27796 = SAUCC 3249C-3.

##### Description.

Conidiophores arising from aerial mycelium, 17–21 μm long, unbranched, reduced to single phialidic pegs, subulate to subcylindrical; aerial conidia hyaline, smooth, and thin-walled, rarely ellipsoidal to falcate, straight to curved dorsiventrally, a blunt apical cell and barely notched basal cell, 1–3(–5)-septate; 1-septate conidia: (12–)12–16(–28) × 3–5 μm (av. 17 × 3 μm, n = 18); 2-septate conidia: (16–)17–21 (–26) × 3–5 μm (av. 19 × 4 μm, n = 17); 3-septate conidia: (20–)22–28 (–36) × 3–6 μm (av. 26 × 4 μm, n = 31); 4-septate conidia: (28–)31–34 (–39) × 4–5 μm (av. 33 × 5 μm, n = 14); 5-septate conidia: (23–)32–33 (–36) × 4–5 μm (av. 31 × 4 μm, n = 5). Sporodochia salmon to saffron, formed abundantly on surface of carnation leaves. Sporodochial conidiophores densely and bearing apical whorls of 1 phialide; sporodochial phialides subulate to subcylindrical, 9–11 × 3–4 μm, smooth, thin-walled, with inconspicuous periclinal thickening; sporodochial conidia falcate, straight to curved dorsiventrally, tapering towards both ends, with slightly papillate, a conical to slightly papillate apical cell, a notched to foot-like basal cell, (0–)1–3(–5)-septate, hyaline, smooth, and thin-walled; 0-septate conidia: (10–)15–20(–21) × 2–4 μm (av. 16 × 3 μm, n = 9); 1-septate conidia: (13–)15–22(–25) × 2–5 μm (av. 18 × 4 μm, n = 23); 2-septate conidia: (13–)16–18(–23) × 2–5 μm (av. 18 × 3 μm, n = 23); 3-septate conidia: (19–)20–25(–29) × 3–5 μm (av. 24 × 4 μm, n = 37); 4-septate conidia: (28–)31–34(–36) × 4–5 μm (av. 33 × 4 μm, n = 12); 5-septate conidia: (34–)34–36(–38) × 3–5 μm (av. 35 × 4 μm, n = 5). Chlamydospores not observed.

**Figure 3.**
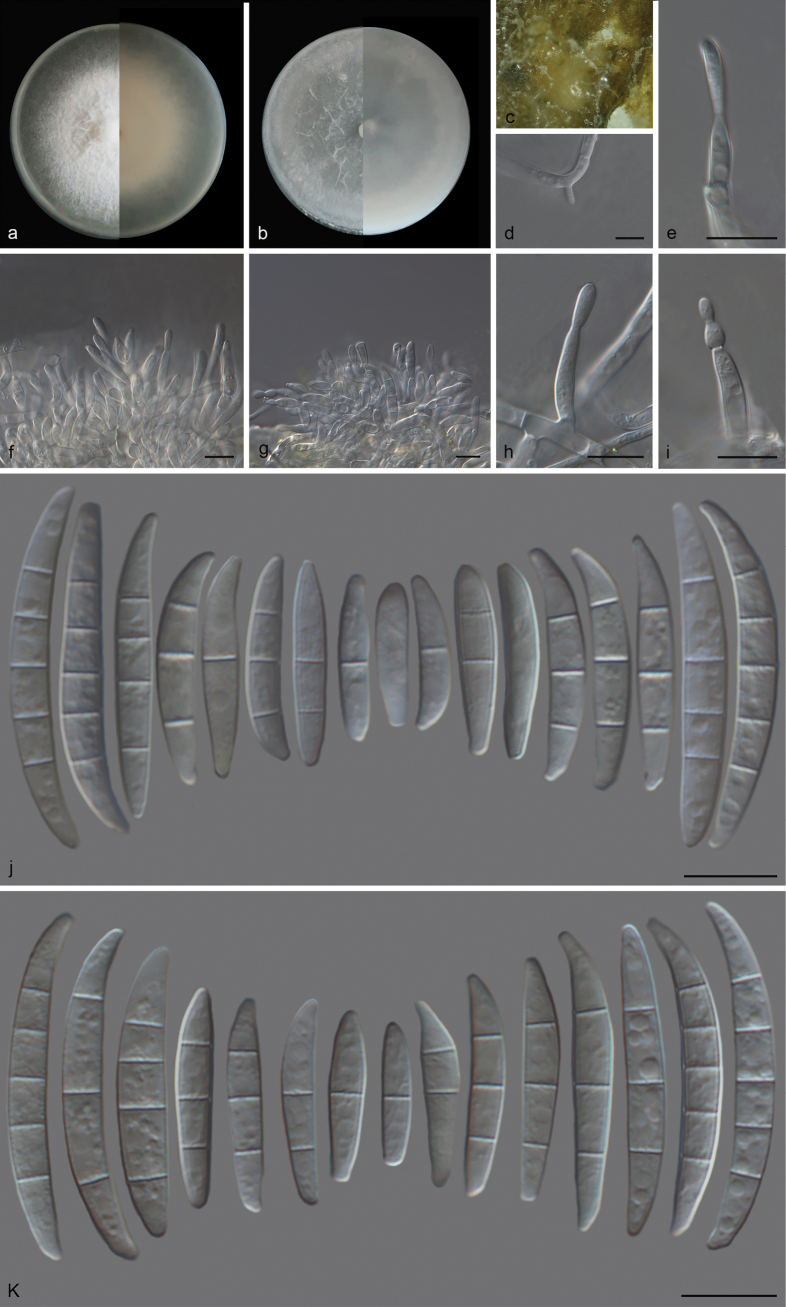
*Fusariumfici* (CGMCC 3.27796) **a** colony on PDA after 7 days at 25 °C (left: above, right: reverse) **b** colony on OA after 7 days at 25 °C (left: above, right: reverse) **c** sporodochia on carnation leaves **d** lateral phialidic peg on aerial mycelium **e** monophialide **f**, **g** sporodochial conidiophores **h**, **i** monophialides on aerial mycelium **j** sporodochial conidia **k** aerial conidia. Scale bars: 10 μm (**d–k**).

##### Culture characteristics.

Colonies on PDA incubated at 25 °C in the dark, reaching 76–80 mm diameter in 7 d, flat, convex, with abundant aerial mycelium, colony margin lightly erose; surface white, odor absent; reverse yellowish white, odor absent. On OA in the dark, reaching 85–90 mm diameter in 7 d; aerial mycelium scant in the center forming a vacant circle, reverse white, odor absent.

##### Additional material studied.

China • Hainan Province, Baoting Li and Miao Autonomous County, on leaves of *Ficusfistulosa*, 10 April 2023, Q.Y. Liu (HSAUP44932), living culture CGMCC 3.27797 = SAUCC 3249C-4.

##### Notes.

Phylogenetic analyses of three combined sequences (*cal*, *rpb2*, and *tef1*) showed that *F.fici* constitutes a distinct clade, closely related to *F.aberrans*. Between *F.fici* (CGMCC 3.27796) and *F.aberrans* (CBS 131385), there were 11/535 differences in *cal*, 13/657 in *rpb2*, and 34/462 in *tef1*. The mycelium on OA of *F.fici* (CGMCC 3.27796) is sparser than that of *F.aberrans* (CBS 131385). Morphologically, *F.fici* (CGMCC 3.27796) and *F.aberrans* (CBS 131385) have different sporodochial conidial septa (0–5-septate in *F.fici* vs. 1–3-septate in *F.aberrans*) and sporodochial phialides (1 phialide in *F.fici* vs. 2–3 phialides in *F.aberrans*). The aerial conidiophores of *F.aberrans* (16–110 μm) are longer than *F.fici* (17–21 μm) (Xia et al. 2019).

#### ﻿ Fusarium weifangense

Taxon classificationFungiHypocrealesNectriaceae

S.L. Han, M.M. Wang & L. Cai, Studies in Mycology 104: 87–148. 2023.

9D4F6CAE-8BC6-57D3-9720-020FD1C674C5

Fig. 4

##### Synonym.

*Fusariumcaulendophyticum* H. Zhang & Y.L. Jiang, Mycosphere 14(1): 2092–2207. 2023.

##### Description.

Conidiophores arising from aerial mycelium, 14–18 μm long, unbranched or irregularly branched, often reduced to single phialides; aerial phialides monophialidic, subulate to subcylindrical, smooth- and thin-walled, with inconspicuous or absent periclinal thickening, 9.2–12.2 × 4.0–4.4 μm; aerial conidia hyaline, rarely ellipsoidal to falcate, slightly curved with almost parallel sides, tapering towards both ends, with a blunt to conical and slightly curved apical cell, blunt to barely notched basal cell, smooth and thin-walled, (1–)3–5-septate; 1-septate conidia: (14–)15–19(–20) × 3–4 μm (av. 17 × 3 μm, n = 8); 2-septate conidia: (19–)19–21(–24) × 3–5 μm (av. 21 × 4 μm, n = 14); 3-septate conidia: (22–)26–31(–34) × 3–6 μm (av. 28 × 4 μm, n = 22); 4-septate conidia: (30–)35–36(–45) × 3–6 μm (av. 36 × 5 μm, n = 17); 5-septate conidia: (31–)34–37(–46) × 4–6 μm (av. 38 × 5 μm, n = 15). Sporodochia salmon to orange, formed abundantly on surface of carnation leaves. Sporodochial conidiophores densely, bearing apical whorls of one phialide; sporodochial phialides monophialidic, subulate to subcylindrical, 16–24 × 2–3 μm, smooth. Sporodochial conidia falcate, slightly curved, tapering towards both ends, with a slightly elongated conical or whip-like curved apical cell, a foot-like to notched basal cell, (0–)4–5-septate, hyaline, thin, and smooth-walled; 0-septate conidia: 26–28 × 3–4 μm; 1-septate conidia: (17–)26–36(–37) × 3–6 μm (av. 28 × 4 μm, n = 10); 2-septate conidia: (20–)21–37 × 3–5 μm (av. 25 × 4 μm, n = 7); 3-septate conidia: 21–33(–38) × 3–5 μm (av. 32 × 5 μm, n = 12); 4-septate conidia: (31–)32–35(–44) × 3–6 μm (av. 36 × 4 μm, n = 22); 5-septate conidia: (34–)40–45(–48) × 3–6 μm (av. 42 × 4 μm, n = 16). Chlamydospores not observed.

**Figure 4.**
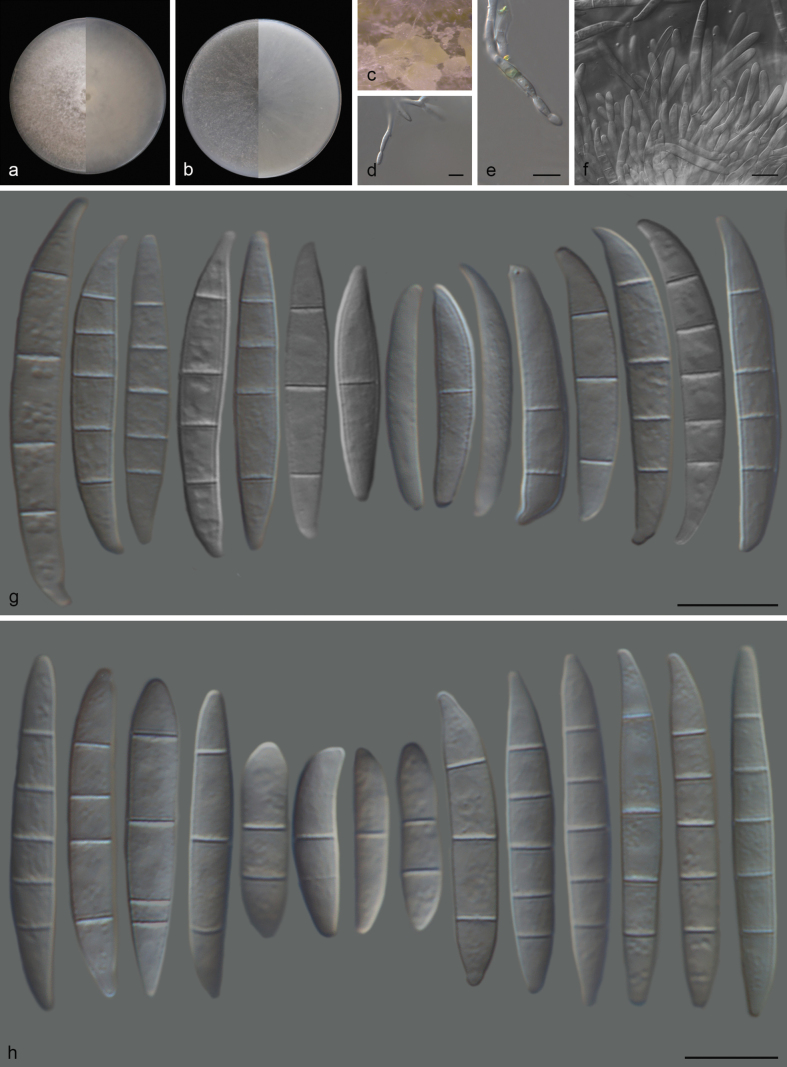
*Fusariumweifangense* (SAUCC 5208C-2) **a** colony on PDA after 7 days at 25 °C (left: above, right: reverse) **b** colony on OA after 7 days at 25 °C (left: above, right: reverse) **c** sporodochia on carnation leaves **d** polyphialide **e** monophialide **f** sporodochial conidiophores **g** sporodochial conidia **h** aerial conidia. Scale bars: 10 μm (**d–h**).

##### Culture characteristics.

Colonies on PDA incubated at 25 °C in the dark, reaching 86–90 mm diameter in 7 d; surface white, flat, felty to velvety, aerial mycelia dense, colony margin entire; reverse white, odor absent. Colonies on OA incubated at 25 °C in the dark, reaching 85–89 mm diameter in 7 d; surface white and aerial mycelia scant, radiate, reverse white, radiate, odor absent.

##### Materials examined.

China • Sichuan Province, Baoting Li and Miao Autonomous County, on leaves of *Prunussalicina*, 2 July 2023, Q.Y. Liu (HSAUP20852, HSAUP30852), living cultures SAUCC 5208C-2 = CGMCC 3.27939, SAUCC 5208C-3.

##### Notes.

*Fusariumweifangense* (LC18333, ex-type strain) was proposed by Han et al. (2023). *Fusariumcaulendophyticum* (CGMCC 3.25474, ex-type strain) was proposed by Zhang et al. (2023a). *Fusariumweifangense* (LC18333, ex-type strain) was the first to be discovered. *Fusariumweifangense* (LC18333 and LC18243) are clustered with *Fusariumcaulendophyticum* (CGMCC 3.25474 and GUCC 191050.2) clade in the combined phylogenetic tree (Fig. 1, Suppl. material 4). *Fusariumweifangense* (LC18333, ex-type strain) and *Fusariumcaulendophyticum* (CGMCC 3.25474, ex-type strain) were similar in *cal* (0/535), *rpb2* (1/657), and *tef1* (2/462) sequences. We therefore considered the *Fusariumcaulendophyticum* synonym of *Fusariumweifangense*. In this study, our strains (SAUCC 5208C-2 and SAUCC 5208C-3) are clustered with the *Fusariumweifangense* (LC18333 and LC18243) clade in the combined phylogenetic tree (Fig. 1). SAUCC 5208C-2 and SAUCC 5208C-3 were similar to the latter in *cal* (with 100% sequence identity), *rpb2* (99.85%), and *tef1* (98.70%) sequences. *Fusariumweifangense* was previously isolated from wheat, *Capsicum* sp., *Triticum* sp., *Medicagosativa*, *Lactucasativa*, *Chenopodiumquinoa*, and *Rosaceaeroxburghii*, and it has now been reported for the first time on *Prunussalicina* (Wang et al. 2019; Xia et al. 2019; Yin et al. 2021; Han et al. 2023; Zhang et al. 2023a) (Suppl. material 3).

#### ﻿ Fusarium xylosmatis

Taxon classificationFungiHypocrealesNectriaceae

Q.Y. Liu, X.G. Zhang & J.W. Xia sp. nov.

D333CA4A-0112-5AA3-AD32-47596A40F84F

MycoBank No: 856642

Fig. 5

##### Etymology.

Referring to the genus name of the host plant *Xylosmacongesta*.

##### Typus.

China • Yunan Province, Nanuo Mountain, on leaves of *Xylosmacongesta*, 3 March 2023, Q.Y. Liu (HMAS 353394, holotype), ex-holotype culture CGMCC 3.27794 = SAUCC 2416-1.

##### Description.

Conidiophores arising from aerial mycelium, 25–35 μm long, unbranched or irregularly branched, often reduced to single phialides, subulate to subcylindrical, smooth, 12–15 × 4–5 μm, periclinal thickening inconspicuous; aerial conidia ellipsoidal to falcate, slightly curved, tapering towards both ends, with a blunt to conical and slightly curved apical cell and papillate basal cell, (0–)3–5-septate; 0-septate conidia: 16–20 × 3–4 μm (av. 21 × 4 μm, n = 5); 1-septate conidia: (12–)15–19(–29) × 3–4 μm (av. 18 × 4 μm, n = 33); 2-septate conidia: (16–)16–23(–29) × 3–5 μm (av. 21 × 4 μm, n = 18); 3-septate conidia: (20–)30–36(–41) × 4–5 μm (av. 31 × 5 μm, n = 45); 4-septate conidia: (31–)30–36(–34) × 4–6 μm (av. 34 × 5 μm, n = 26); 5-septate conidia: (30–)37–41(–43) × 4–6 μm (av. 38 × 5 μm, n = 26). Sporodochia pale orange, formed abundantly on surface of carnation leaves. Sporodochial conidiophores densely and irregularly branched, 15–19 × 2–3 μm, bearing apical whorls of 1–2 phialides; sporodochial phialides monophialidic, subulate to subcylindrical, 10–12 × 2–3 μm, smooth, and thin-walled; sporodochial conidia falcate, curved dorsiventrally, straight to slightly curved, tapering towards both ends, with slightly papillate, curved apical cell and a notched to foot-like basal cell, (0–)3–4(–5)-septate, hyaline, smooth, and thin-walled; 0-septate conidia: 28–30 × 3–4 μm (av. 29 × 4 μm, n = 5); 1-septate conidia: (16–)21–32(–36) × 3–5 μm (av. 27 × 4 μm, n = 11); 2-septate conidia: 22–23 × 3–4 μm (av. 23 × 4 μm, n = 4); 3-septate conidia: (22–)25–33(–41) × 3–6 μm (av. 32 × 4 μm, n = 38); 4-septate conidia: (33–)35–38(–43) × 4–6 μm (av. 37 × 5 μm, n = 26); 5-septate conidia: (36–)38–40(–44) × 4–6 μm (av. 40 × 5 μm, n = 16). Chlamydospores abundant, globose, subglobose to ellipsoid, terminal or intercalary, solitary, in pairs, or forming long chains, 8–12 μm diameter.

**Figure 5.**
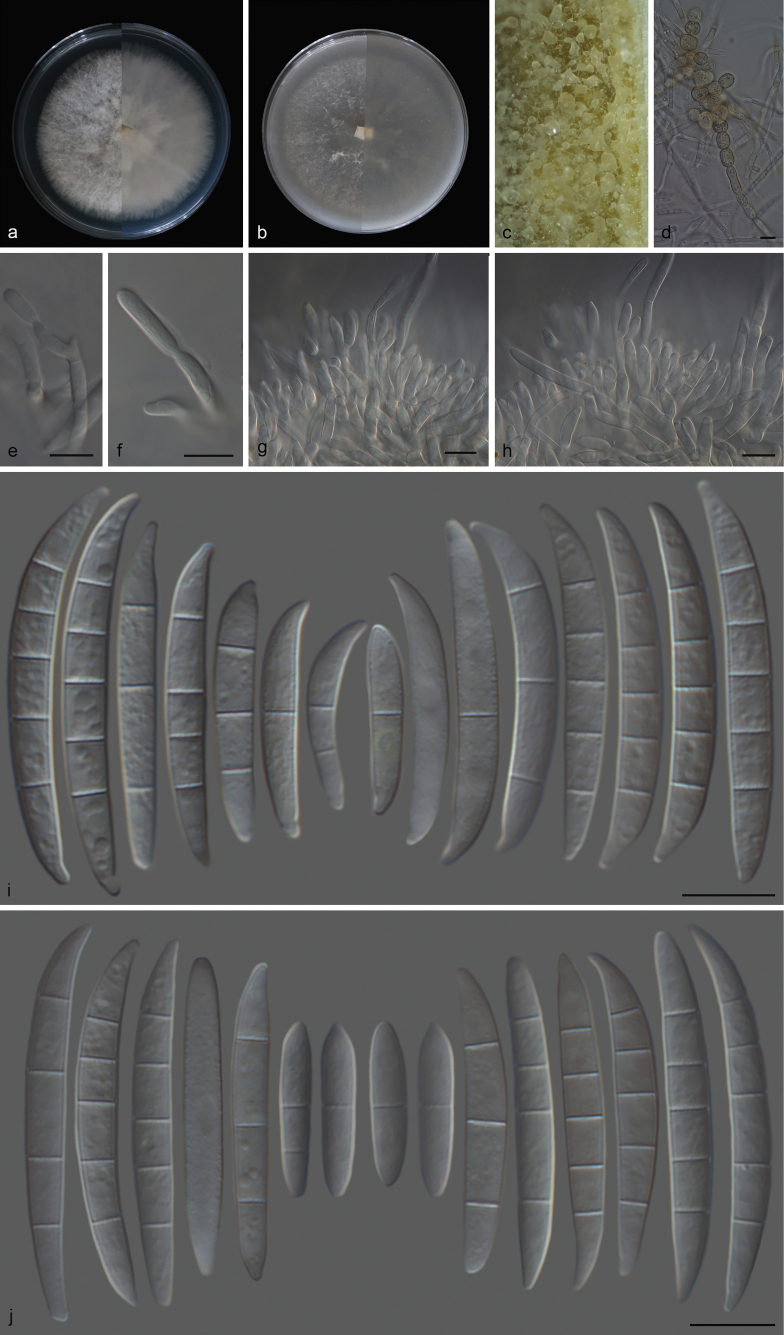
*Fusariumxylosmatis* (CGMCC 3.27794) **a** colony on PDA after 7 days at 25 °C (left: above, right: reverse) **b** colony on OA after 7 days at 25 °C (left: above, right: reverse) **c** sporodochia on carnation leaves **d** chlamydospores **e** polyphialide **f** monophialide **g, h** sporodochial conidiophores **i** sporodochial conidia **j** aerial conidia. Scale bars: 10 μm (**d–j**).

##### Culture characteristics.

Colonies on PDA incubated at 25 °C in the dark, reaching 71–79 mm diameter in 7 d; aerial mycelia dense, flat, white, colony margin entire; reverse yellowish white, radiate, aerial mycelia dense, odor absent. Colonies on OA grown in the dark, reaching 69–77 mm diameter after 7 d at 25 °C, flat, aerial mycelia scant, colony margin entire, white; reverse white, odor absent.

##### Additional material studied.

China • Yunan Province, Nanuo Mountain, on leaves of *Xylosmacongesta*, 3 March 2023, Q.Y. Liu (HSAUP21624), living culture CGMCC 3.27795 = SAUCC 2416-2.

##### Notes.

Phylogenetically, *F.xylosmatis* (CGMCC 3.27794) is closely related to the species *F.weifangense* (LC18333); there were 7/535 differences in *cal*, 9/657 in *rpb2*, and 8/462 in *tef1*. Morphologically, *F.xylosmatis* (CGMCC 3.27794) is distinguished from *F.weifangense* (LC18333) by the number of sporodochial conidial septa (0–5-septate in *F.xylosmatis* (CGMCC 3.27794) vs. 3–7-septate in *F.weifangense* (LC18333)) (Han et al. 2023; Zhang et al. 2023a).

## ﻿Discussion

The genus and species concepts in *Fusarium* have endured significant changes (Leslie and Summerell 2006; Crous et al. 2021; He et al. 2024). Traditionally, the identification of *Fusarium* is mainly based on morphological characteristics (Wollenweber and Reinking 1935; Snyder and Hansen 1940; Toussoun and Nelson 1968; Gerlach and Nirenberg 1982; Leslie and Summerell 2006). However, identification is difficult due to the high morphological variation that complicates morphological identification among the closely related species (Leslie and Summerell 2006; Crous et al. 2021). Therefore, it is important to identify *Fusarium* species through molecular analysis (Wang et al. 2019; Xia et al. 2019; Crous et al. 2021; Wang et al. 2022; He et al. 2024). The internal transcribed spacer (ITS), the large subunit (LSU), ATP citrate lyase (*acl1*), calmodulin (*cal*), RNA polymerase II largest subunit (*rpb1*), RNA polymerase second largest subunit (*rpb2*), translation elongation factor 1-alpha (*tef1*), and beta-tubulin (*tub2*) are used in current studies (Lombard et al. 2015; Sandoval-Denis et al. 2018; Xia et al. 2019; Crous et al. 2021; Suwannarach et al. 2023; He et al. 2024). However, the identification of *Fusarium* at the species level could not be resolved using the ribosomal DNA gene (ITS and LSU) alone (Balajee et al. 2009; O’Donnell et al. 2015; Suwannarach et al. 2023). Thus, the protein-coding genes (*acl1*, *cal*, *rpb1*, *rpb2*, *tef1*, and *tub2*) are added (Xia et al. 2019; Crous et al. 2021; Suwannarach et al. 2023; He et al. 2024). Different complexes of *Fusarium* require different gene combinations to identify.

In this study, we collected parasitic or saprotrophic fungi from terrestrial habitats in Hainan, Sichuan, and Yunnan Provinces of China on four plant specimens: *Setariapalmifolia*, *Ficusfistulosa*, *Prunussalicina*, and *Xylosmacongesta*. Morphologically, these species exhibit a range of variations in spore size, shape, and ornamentation, as well as colony characteristics such as growth rate, pigmentation, and texture. We also conducted phylogenetic analyses using *cal*, *rpb2*, and *tef1* sequences and can be recognized as two new phylogenetic species (*Fusarium.fici* sp. nov. and *Fusariumxylosmatis* sp. nov.), along with two known species (*Fusariumfecundum* and *Fusariumweifangense*). The discovery of two new species underscores the rich fungal diversity in Hainan, Sichuan, and Yunnan Provinces and emphasizes the need for further exploration of understudied habitats. *Fusariumfecundum* was first reported from *Setariapalmifolia*; *Fusariumweifangense* was first reported from *Prunussalicina*. It can contribute to our knowledge of host specificity and ecological adaptation in fungal pathogens. These findings have significant implications for fungal taxonomy, ecology, and potential applications in plant pathology and biocontrol.

## Supplementary Material

XML Treatment for Fusarium fecundum

XML Treatment for Fusarium fici

XML Treatment for Fusarium weifangense

XML Treatment for Fusarium xylosmatis

## ﻿Additional information

### Author ORCIDs

Qiyun Liu https://orcid.org/0009-0009-9545-7962

Zhaoxue Zhang https://orcid.org/0000-0002-4824-9716

Duhua Li https://orcid.org/0009-0006-5200-2034

Xiuguo Zhang https://orcid.org/0000-0001-9733-8494

Jiwen Xia https://orcid.org/0000-0002-7436-7249

### Data availability

The sequences were deposited in the GenBank database.

## Supplementary materials

Supplementary material 1 GenBank accession numbers of the taxa used in phylogenetic reconstruction Data type docx https://binary.pensoft.net/file/1301257This dataset is made available under the Open Database License (http://opendatacommons.org/licenses/odbl/1.0/). The Open Database License (ODbL) is a license agreement intended to allow users to freely share, modify, and use this Dataset while maintaining this same freedom for others, provided that the original source and author(s) are credited.Congcong Ai, Qiyun Liu, Yaling Wang, Zhaoxue Zhang, Duhua Li, Yun Geng, Xiuguo Zhang, Jiwen Xia

Supplementary material 2 Phylogeny of the *Fusariumincarnatum-equiseti* species complex (FIESC) inferred based on the *cal* (a), *rpb2* (b), and *tef1* (c) loci, respectively Data type docx https://binary.pensoft.net/file/1301258This dataset is made available under the Open Database License (http://opendatacommons.org/licenses/odbl/1.0/). The Open Database License (ODbL) is a license agreement intended to allow users to freely share, modify, and use this Dataset while maintaining this same freedom for others, provided that the original source and author(s) are credited.Congcong Ai, Qiyun Liu, Yaling Wang, Zhaoxue Zhang, Duhua Li, Yun Geng, Xiuguo Zhang, Jiwen Xia

Supplementary material 3 GenBank accession numbers of the taxa used in phylogenetic reconstruction (Suppl. material 4) Data type docx https://binary.pensoft.net/file/1301259This dataset is made available under the Open Database License (http://opendatacommons.org/licenses/odbl/1.0/). The Open Database License (ODbL) is a license agreement intended to allow users to freely share, modify, and use this Dataset while maintaining this same freedom for others, provided that the original source and author(s) are credited.Congcong Ai, Qiyun Liu, Yaling Wang, Zhaoxue Zhang, Duhua Li, Yun Geng, Xiuguo Zhang, Jiwen Xia

Supplementary material 4 Phylogeny inferred based on the combined *cal*-*rpb2*-*tef1* sequence dataset with *Fusariumconcolor* (NRRL 13459) as the outgroup Data type docx https://binary.pensoft.net/file/1301260This dataset is made available under the Open Database License (http://opendatacommons.org/licenses/odbl/1.0/). The Open Database License (ODbL) is a license agreement intended to allow users to freely share, modify, and use this Dataset while maintaining this same freedom for others, provided that the original source and author(s) are credited.Congcong Ai, Qiyun Liu, Yaling Wang, Zhaoxue Zhang, Duhua Li, Yun Geng, Xiuguo Zhang, Jiwen Xia
