# Supplementary material for: ﻿Morphological and phylogenetic analyses reveal new species and records of Fusarium (Nectriaceae, Hypocreales) from China
Source: MycoKeys. 2025 Apr 7;116:53–71. doi: 10.3897/mycokeys.116.150363 (PMC11997610; doi:10.3897/mycokeys.116.150363)
Supplement: Supplementary material 1 — GenBank accession numbers of the taxa used in phylogenetic reconstruction [file mycokeys-116-053-s001.docx]

**Supplementary material 1.** GenBank accession numbers of the taxa used in phylogenetic reconstruction.

| **Species** | **Culture accession** | **Host/substrate** | **Location** | **GenBank accession numbers** | | |
| --- | --- | --- | --- | --- | --- | --- |
|  |  |  |  | ***cal*** | ***rpb2*** | ***tef1*** |
| *Fusarium aberrans* | CBS 119866 = MRC 6715 | Sorghum malt | Niger | MN170310 | MN170377 | MN170444 |
| *F. aberrans* | CBS 131385^T^ | *Oryza australiensis* | Australia | MN170311 | MN170378 | MN170445 |
| *F. aberrans* | CBS 131387 | *Oryza australiensis* | Australia | MN170312 | MN170379 | MN170446 |
| *F. aberrans* | CBS 131388 | *Oryza australiensis* | Australia | MN170313 | MN170380 | MN170447 |
| *F. arcuatisporum* | NRRL 32997 | Human toenail | USA | GQ505536 | GQ505802 | GQ505624 |
| *F. brevicaudatum* | NRRL 43638^T^ | *Trichechus* sp. | USA | GQ505576 | GQ505843 | GQ505665 |
| *F. brevicaudatum* | NRRL 43694 | Human eye | USA | GQ505579 | GQ505846 | GQ505668 |
| *F. brevicaudatum* | NRRL 45998 | Human toe | USA | GQ505584 | GQ505851 | GQ505673 |
| *F. bubalinum* | CBS 161.25 = NRRL 26857 = NRRL 26918^T^ | Unknown | Australia | MN170314 | MN170381 | MN170448 |
| *F. caatingaense* | CBS 976.97 | *Juniper chinensis* | USA | MN170315 | MN170382 | MN170449 |
| *F.* *caatingaense* | NRRL 34003 = CBS 130317 | Human sputum | USA | GQ505539 | GQ505805 | GQ505627 |
| *F. camptoceras* | CBS 193.65 = ATCC 16065 =  BBA 9810 = IMI 112500^ET^ | *Theobroma cacao* | Costa Rica | MN170316 | MN170383 | MN170450 |
| *F. cateniforme* | CBS 150.25 = ATCC 11853^T^ | Unknown | Unknown | MN170317 | MN170384 | MN170451 |
| *F. caulicola* | GUCC 191051.1 = CGMCC 3.25475^T^ | *Rosa roxburghii* | China | OR043733 | OR043828 | OR043883 |
| *F. caulicola* | GUCC 191051.2 | *Rosa roxburghii* | China | OR043734 | OR043829 | OR043884 |
| *F. citri* | LC6896 = CGMCC 3.19467^T^ | Citrus reticulata leaf | Hunan | MK289668 | MK289771 | MK289617 |
| *F.* *citri* | NRRL 52765 = ARSEF 2304 | *Heteropsylla* | Papua New Guinea | − | JF741165 | JF740839 |
| *F. citrullicola* | SDBR-CMU422^T^ | *Citrullus lanatus* | Thailand | OP020924 | OP020928 | OP020920 |
| *F. citrullicola* | SDBR-CMU423 | *Citrullus lanatus* | Thailand | OP020925 | OP020929 | OP020921 |
| *F. clavum* | CBS 394.93 = BBA 64265 = NRRL 25795 | *Disphyma crassifolium* | Germany | GQ505509 | GQ505775 | GQ505597 |
| *F. clavum* | CBS 126202^T^ | Soil | Namibia | MN170322 | MN170389 | MN170456 |
| *F. clavum* | CBS 119881 = MRC 8412 | Unknown | Unknown | MN170323 | MN170390 | MN170457 |
| *F. clavum* | NRRL 32871 = FRC R-9561 | Human abscess | USA | GQ505531 | GQ505797 | GQ505619 |
| *F. clavum* | NRRL 34032 | Mandibular abscess | USA | GQ505547 | GQ505813 | GQ505635 |
| *F. clavum* | NRRL 34035 | Human sinus cavity | USA | GQ505549 | GQ505815 | GQ505637 |
| *F. coffeatum* | CBS 635.76 = BBA 62053 = NRRL 20841^T^ | *Cynodon lemfuensis* | New Zealand | MN120696 | MN120736 | MN120755 |
| *F. coffeatum* | NRRL 28577 = CBS 430.81 | Grave stone | Romania | MN120697 | MN120737 | MN120756 |
| *F. compactum* | CBS 185.31 = NRRL 36318 | Unknown | Unknown | GQ505558 | GQ505824 | GQ505646 |
| *F. compactum* | CBS 186.31 = NRRL 36323^ET^ | Cotton yarn | England | GQ505560 | GQ505826 | GQ505648 |
| *F. concolor* | NRRL 13459 = ATCC 60096 = CBS 961.87 = FRC M-2405 = IMI 296456^T^ | Plant debris | South Africa | GQ505585 | GQ505852 | GQ505674 |
| *F. croceum* | CBS 131777^T^ | *Triticum* sp. | Iran | MN170329 | MN170396 | MN170463 |
| *F. croceum* | CBS 131788 | *Triticum* sp. | Iran | MN170330 | MN170397 | MN170464 |
| *F. croceum* | CPC 35240 | Soil | Czech Republic | MN170331 | MN170398 | MN170465 |
| *F. croceum* | NRRL 3020 = FRC R-6053 = MRC 2231 | Unknown | Unknown | GQ505498 | GQ505764 | GQ505586 |
| *F. croceum* | NRRL 3214 = FRC R-6054 = MRC 2232 | Unknown | Unknown | GQ505499 | GQ505765 | GQ505587 |
| *F. duofalcatisporum* | CBS 264.50 = NRRL 36401 | *Gossypium hirsutum* | Mozambique | GQ505563 | GQ505829 | GQ505651 |
| *F.* *duofalcatisporum* | CBS 384.92 = NRRL 36448^T^ | *Phaseolus vulgaris* | Sudan | GQ505564 | GQ505830 | GQ505652 |
| *F. equiseti* | CBS 414.86 = FRC R-8508 = IMI 309348 | Potato peel | Denmark | MN170333 | MN170400 | MN170467 |
| *F. equiseti* | CBS 119663 | Maize husk | Switzerland | MN170334 | MN170401 | MN170468 |
| *F. equiseti* | CPC 35220 | Sediment | Czech Republic | MN170337 | MN170404 | MN170471 |
| *F. extenuatum* | LLC1492 | Sorghum | Ethiopia | OP486038 | OP486727 | OP487157 |
| *F. extenuatum* | LLC1501^T^ | Sorghum | Ethiopia | OP486039 | OP486728 | OP487158 |
| *F. fasciculatum* | CBS 131382^T^ | *Oryza australiensis* | Australia | MN170339 | MN170406 | MN170473 |
| *F. fasciculatum* | CBS 131383 | *Oryza australiensis* | Australia | MN170340 | MN170407 | MN170474 |
| *F. fasciculatum* | CBS 131384 | *Oryza australiensis* | Australia | MN170341 | MN170408 | MN170475 |
| *F. fecundum* | LC15875 = HSL1587 = CGMCC 3.23516^T^ | Wheat | China | OQ125281 | OQ125544 | PV008845 |
| *F. fecundum* | LC18376 = HSL197 | Rice | China | OQ125280 | OQ125543 | PV008846 |
| ***F. fecundum*** | **SAUCC 2414-4** = **CGMCC 3.27792** | ***Setaria palmifolia*** | **China** | **PQ309113** | **PQ309121** | **PV008845** |
| ***F. fecundum*** | **SAUCC 2414-5** = **CGMCC 3.27793** | ***Setaria palmifolia*** | **China** | **PQ309114** | **PQ309122** | **PV008846** |
| ***F. fici*** | **SAUCC 3249C-3 = CGMCC 3.27796 ^T^** | ***Ficus fistulosa*** | **China** | **PQ309111** | **PQ309123** | **PQ309133** |
| ***F. fici*** | **SAUCC 3249C-4 = CGMCC 3.27797** | ***Ficus fistulosa*** | **China** | **PQ309112** | **PQ309124** | **PQ309134** |
| *F. flagelliforme* | CBS 162.57 = NRRL 36269^T^ | *Pinus nigra* | Croatia | GQ505557 | GQ505823 | GQ505645 |
| *F. flagelliforme* | CBS 259.54 = NRRL 36392 | Unknown seedling | Germany | GQ505562 | GQ505828 | GQ505650 |
| *F. flagelliforme* | NRRL 26921 = CBS 731.87 | *Triticum* sp. | Germany | GQ505512 | GQ505778 | GQ505600 |
| *F. flagelliforme* | NRRL 31011 = BBA 69079 | *Thuja* sp. | Germany | GQ505518 | GQ505784 | GQ505606 |
| *F. gracilipes* | NRRL 43635^T^ | Horse | USA | GQ505573 | GQ505840 | GQ505662 |
| *F. guilinense* | NRRL 13335 = FRC R-2138 | Alfalfa | Australia | GQ505502 | GQ505768 | GQ505590 |
| *F. guilinense* | NRRL 32865 = FRC R-8480 | Human endocarditis | Brazil | GQ505526 | GQ505792 | GQ505614 |
| *F. hainanense* | NRRL 26417 = CBS 544.96 | Leaf litter | Cuba | GQ505510 | GQ505776 | GQ505598 |
| *F. humuli* | LC 4490 | *Osmanthus* sp. | China | MK289664 | MK289767 | MK289614 |
| *F. humuli* | LC 12158 | *Musa nana* | China | MK289645 | MK289745 | MK289592 |
| *F. humuli* | LC 12159 | *Musa nana* | China | MK289646 | MK289746 | MK289593 |
| *F. incarnatum* | NRRL 32866 = FRC R-8822 | Human | USA | GQ505527 | GQ505793 | GQ505615 |
| *F. ipomoeae* | CBS 135762 | *Miscanthus giganteus* | USA | MN170344 | MN170411 | MN170478 |
| *F. ipomoeae* | CBS 140909 | *Solanum lycopersicum* | Russia | MN170345 | MN170412 | MN170479 |
| *F. ipomoeae* | NRRL 34039 | Human | USA | GQ505551 | GQ505817 | GQ505639 |
| *F. irregulare* | NRRL 31160 | Human lung | USA | GQ505519 | GQ505785 | GQ505607 |
| *F. irregulare* | NRRL 32182 | Human blood | USA | GQ505523 | GQ505789 | GQ505611 |
| *F. irregulare* | NRRL 32869 = FRC R-9445 | Human | USA | GQ505530 | GQ505796 | GQ505618 |
| *F. jinanense* | LC15878 = HSL751 = CGMCC 3.23519^T^ | Maize | China | OQ125271 | OQ125521 | OQ125131 |
| *F. jinanense* | LC18379 = HSL1983 | Maize | China | OQ125272 | OQ125522 | OQ125132 |
| *F. kotabaruense* | InaCC F963^T^ | *Musa* sp. | Indonesia | LS479429 | LS479859 | LS479445 |
| *F. lacertarum* | NRRL 20423 = ATCC 42771 =  CBS 130185 = IMI 300797^T^ | Lizard skin | India | GQ505505 | GQ505771 | GQ505593 |
| *F. lacertarum* | NRRL 36123 = CBS 102300 | Unknown | Unknown | GQ505555 | GQ505821 | GQ505643 |
| *F. longicaudatum* | CBS 123.73 = ATCC 24370  = IMI 160825 = NRRL 25477^T^ | Unknown | Tanzania | MN170347 | MN170414 | MN170481 |
| *F. longifundum* | CBS 235.79 = NRRL 36372^T^ | Air | Netherlands | GQ505561 | GQ505827 | GQ505649 |
| *F. luffae* | CBS 131097 | *Setaria verticilata* | Iran | MN170348 | MN170415 | MN170482 |
| *F. luffae* | NRRL 31167 | Human sputum | USA | GQ505520 | GQ505786 | GQ505608 |
| *F. luffae* | NRRL 32522 | Human diabetic cellulitis | USA | GQ505524 | GQ505790 | GQ505612 |
| *F. mianyangense* | LC15879 = HSL859 = CGMCC 3.23520^T^ | Rice | China | OQ125335 | OQ125510 | OQ125232 |
| *F. monophialidicum* | NRRL 54973 | Rhinoceros eye | USA | MN170349 | MN170416 | MN170483 |
| *F. mucidum* | CBS 102394 | *Anacardium occidentale* | El Salvador | MN170350 | MN170417 | MN170484 |
| *F. mucidum* | CBS 102395^T^ | *Anacardium occidentale* | El Salvador | MN170351 | MN170418 | MN170485 |
| *F.* *mucidum* | Indo 175 | *Musa acuminata* | Indonesia | LS479431 | LS479862 | LS479447 |
| *F. multiceps* | CBS 130386 = NRRL 43639^T^ | *Trichechus* sp. | USA | GQ505577 | GQ505844 | GQ505666 |
| *F. nanum* | CBS 119867 = FRC R-4237 = MRC 3228 | *Sorghum* sp. | Unknown | MN170352 | MN170419 | MN170486 |
| *F. nanum* | CBS 131781 | *Triticum* sp. | Iran | MN170353 | MN170420 | MN170487 |
| *F. nanum* | NRRL 32993 | Human nasal tissue | USA | GQ505532 | GQ505798 | GQ505620 |
| *F. neoscirpi* | CBS 610.95 = NRRL 26861 = NRRL 26922^T^ | Soil | France | GQ505513 | GQ505779 | GQ505601 |
| *F. neosemitectum* | CBS 189.60^T^ | *Musa sapientum* | Dem. Rep. Congo | MN170355 | MN170422 | MN170489 |
| *F. neosemitectum* | CBS 190.60 | *Musa sapientum* | Dem. Rep. Congo | MN170356 | MN170423 | MN170490 |
| *F. nothincarnatum* | LC18382 = HSL199 | Wheat | China | OQ125289 | OQ125508 | OQ125146 |
| *F. nothincarnatum* | LC18436 = HSL221 = CGMCC 3.24286^T^ | Rice | China | OQ125290 | OQ125509 | OQ125147 |
| *F. pernambucanum* | CBS 132194 | Human ﬁnger nail | Thailand | MN170358 | MN170425 | MN170492 |
| *F. pernambucanum* | CBS 133024 | Human foot | Thailand | MN170360 | MN170427 | MN170494 |
| *F. persicinum* | CBS 479.83^T^ | Unknown | Unknown | MN170361 | MN170428 | MN170495 |
| *F. persicinum* | CBS 143596 = CPC 30848 | *Stereum hirsutum* | Iran | LT970732 | LT970751 | LT970779 |
| *F. persicinum* | CBS 143598 = CPC 30850 | Smut | Iran | LT970733 | LT970752 | LT970780 |
| *F. persicinum* | CBS 143600 = CPC 30852 | Smut | Iran | LT970734 | LT970753 | LT970781 |
| *F. persicinum* | CBS 143606 = CPC 30858 | Smut | Iran | LT970736 | LT970755 | LT970783 |
| *F. radicigenum* | GUCC 197371.1 | *Rosa roxburghii* | China | OR043752 | OR043851 | OR043907 |
| *F. radicigenum* | GUCC 197425.1 | *Rosa roxburghii* | China | OR043753 | OR043852 | OR043908 |
| *F. radicigenum* | GUCC 197221.1 = CGMCC 3.25478^T^ | *Rosa roxburghii* | China | OR043754 | – | OR043909 |
| *F. rhinolophicola* | KUMCC 21-0449^T^ | Bat | China | OR022061 | OR025917 | OR026001 |
| *F. rhinolophicola* | KUMCC 21-0450 | Bat | China | OR022063 | OR025919 | OR026003 |
| *F. scirpi* | CBS 447.84 = FRC R-6252 = NRRL 36478^NT^ | Soil | Iran | GQ505566 | GQ505832 | GQ505654 |
| *F. scirpi* | CBS 448.84 = FRC R-6253 | Soil | Australia | MN170364 | MN170431 | MN170498 |
| *F. scirpi* | NRRL 13402 | Soil | Australia | GQ505504 | GQ505770 | GQ505592 |
| *F. serpentinum* | CBS 119880 = BBA 62209 = MRC 1813 | Unknown | Unknown | MN170365 | MN170432 | MN170499 |
| *F. sulawesiense* | InaCC F940^T^ | *Musa acuminata* | Indonesia | LS479422 | LS479855 | LS479443 |
| *F. sulawesiense* | InaCC F941 | *Musa acuminata* | Indonesia | LS479423 | LS479856 | LS479444 |
| *F. tanahbumbuense* | CBS 145.44 = BBA 4095 | Unknown | Unknown | MN170371 | MN170438 | MN170505 |
| *F. tanahbumbuense* | CBS 131009 | *Triticum* sp. | Iran | MN170372 | MN170439 | MN170506 |
| *F. tanahbumbuense* | InaCC F965^T^ | *Musa* sp. | Indonesia | LS479432 | LS479863 | LS479448 |
| *F. tanahbumbuense* | NRRL 34005 | Human eye | USA | GQ505541 | GQ505807 | GQ505629 |
| *F. tanahbumbuense* | NRRL 43297 | Unknown | Unknown | GQ505569 | GQ505835 | GQ505657 |
| *F. tangerinum* | LLC3018 | Sorghum | Ethiopia | OP486066.1 | OP486757.1 | OP487188.1 |
| *F. tangerinum* | LLC3501^T^ | Sorghum | Ethiopia | OP486067.1 | OP486758.1 | OP487189.1 |
| *F. toxicum* | CBS 219.63 | Soil | Germany | MN170373 | MN170440 | MN170507 |
| *F. toxicum* | CBS 406.86 = FRC R-8507 = IMI 309347  = NRRL 25796^T^ | Soil | Germany | MN170374 | MN170441 | MN170508 |
| *F. toxicum* | CBS 130385 | Dog | USA | MN170375 | MN170442 | MN170509 |
| *F. toxicum* | NRRL 43636 | Dog | USA | GQ505574 | GQ505841 | GQ505663 |
| *F. weifangense* | LC18333 = HSL1800 = CGMCC 3.24285^T^ | Wheat | China | OQ125276 | OQ125515 | OQ125107 |
| *F. weifangense* | LC18243 = HSL102 | Wheat | China | OQ125273 | OQ125513 | OQ125106 |
| ***F. weifangense*** | **SAUCC 5208C-2**  = **CGMCC 3.27939** | ***Prunus salicina*** | **China** | **PQ309117** | **PQ309125** | **PQ309127** |
| ***F. weifangense*** | **SAUCC 5208C-3** | ***Prunus salicina*** | **China** | **PQ309118** | **PQ309126** | **PQ309128** |
| *F. weifangense* | GUCC 191050.1 = CGMCC 3.25474 | *Rosa roxburghii* | China | OR043731 | OR043826 | OR043881 |
| *F. weifangense* | GUCC 191050.2 | *Rosa roxburghii* | China | OR043732 | OR043827 | OR043882 |
| *F. wereldwijsianum* | CBS 148219 = NL19-99002 | Soil | Netherlands | MZ921536 | MZ921716 | MZ921848 |
| *F. wereldwijsianum* | CBS 148220 = NL19-99003 | Soil | Netherlands | MZ921537 | MZ921717 | MZ921849 |
| ***F. xylosmatis*** | **SAUCC 2416-1** **= CGMCC 3.27794^T^** | ***Xylosma congesta*** | **China** | **PQ309115** | **PQ309119** | **PQ309131** |
| ***F. xylosmatis*** | **SAUCC 2416-2 = CGMCC 3.27795** | ***Xylosma congesta*** | **China** | **PQ309116** | **PQ309120** | **PQ309132** |
| *Fusarium* sp. | InaCC F964 | *Musa* sp. | Indonesia | LS479425 | LS479860 | LS479446 |
| *Fusarium* sp. | Indo 167 | *Musa* sp. | Indonesia | LS479424 | LS479858 | − |

Ex-type, ex-epitype and ex-neotype strains were indicated in bold with T, ET, and NT, respectively, strains in this study are marked in bold.
