# Supplementary material for: ﻿Morphological and phylogenetic analyses reveal new species and records of Fusarium (Nectriaceae, Hypocreales) from China
Source: MycoKeys. 2025 Apr 7;116:53–71. doi: 10.3897/mycokeys.116.150363 (PMC11997610; doi:10.3897/mycokeys.116.150363)
Supplement: Supplementary material 2 — Phylogeny of the Fusariumincarnatum-equiseti species complex (FIESC) inferred based on the cal (a), rpb2 (b), and tef1 (c) loci, respectively [file mycokeys-116-053-s002.docx]

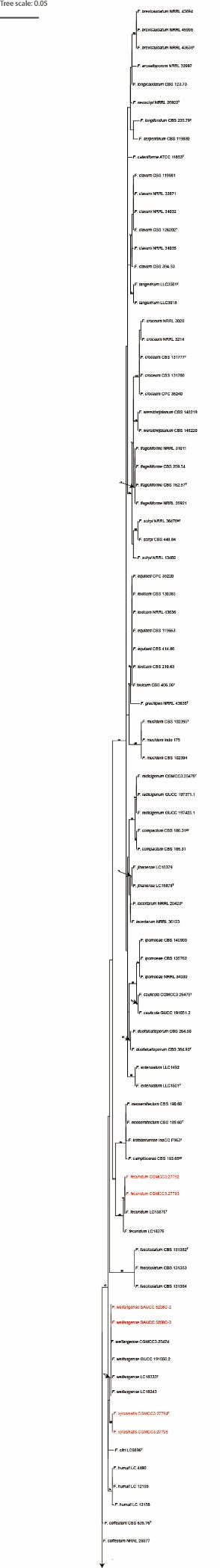

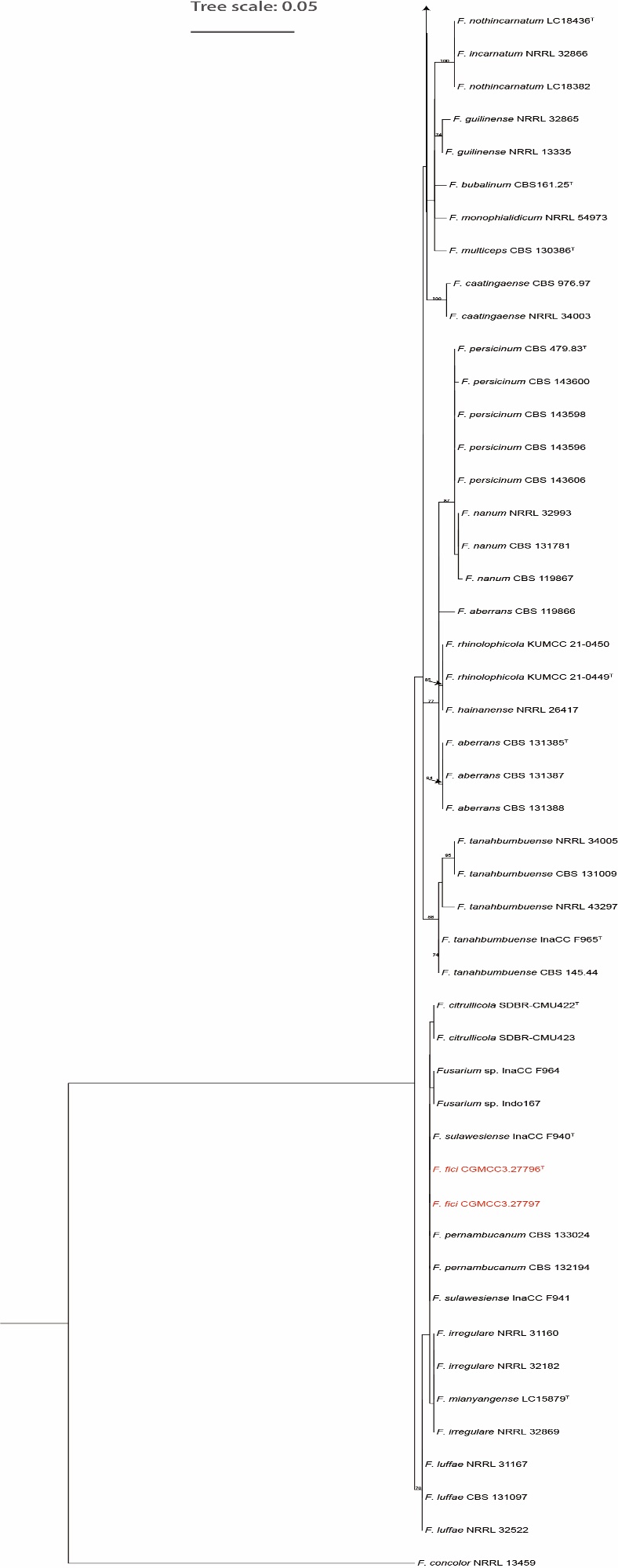


(a)


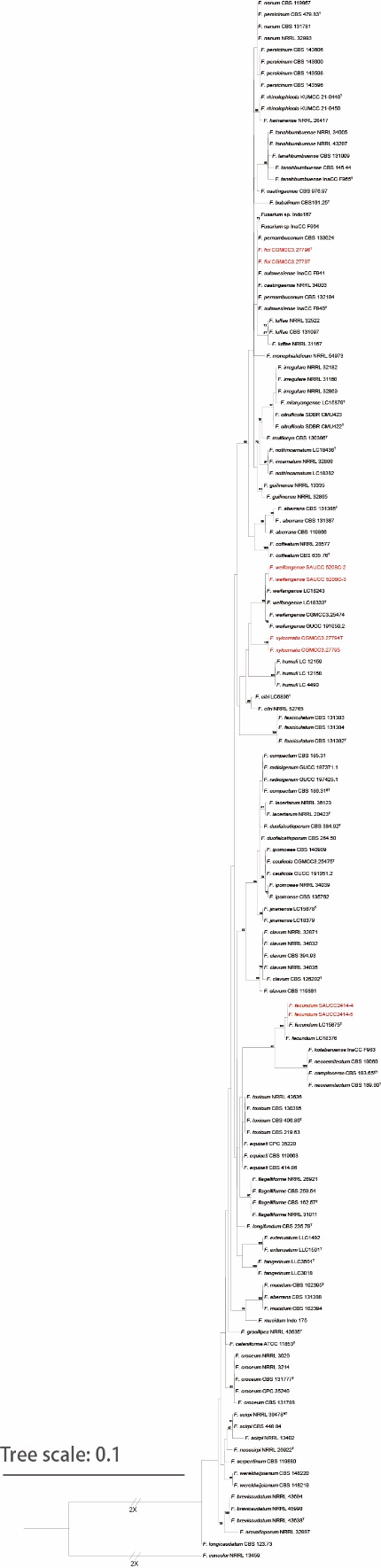


(b)


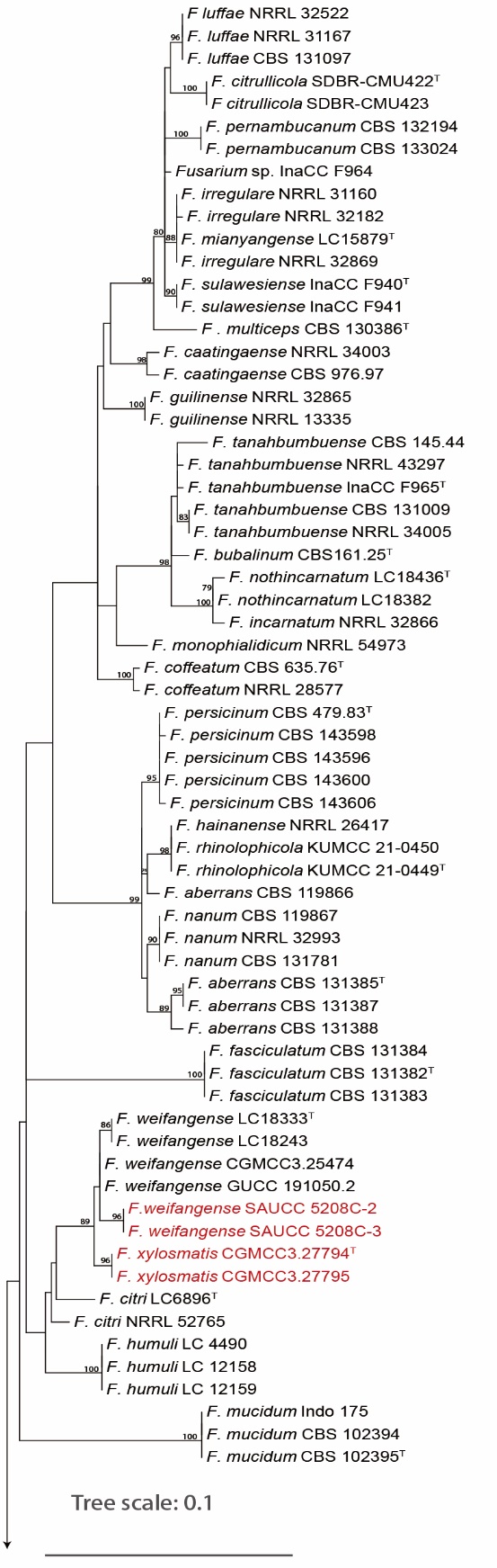


(c)


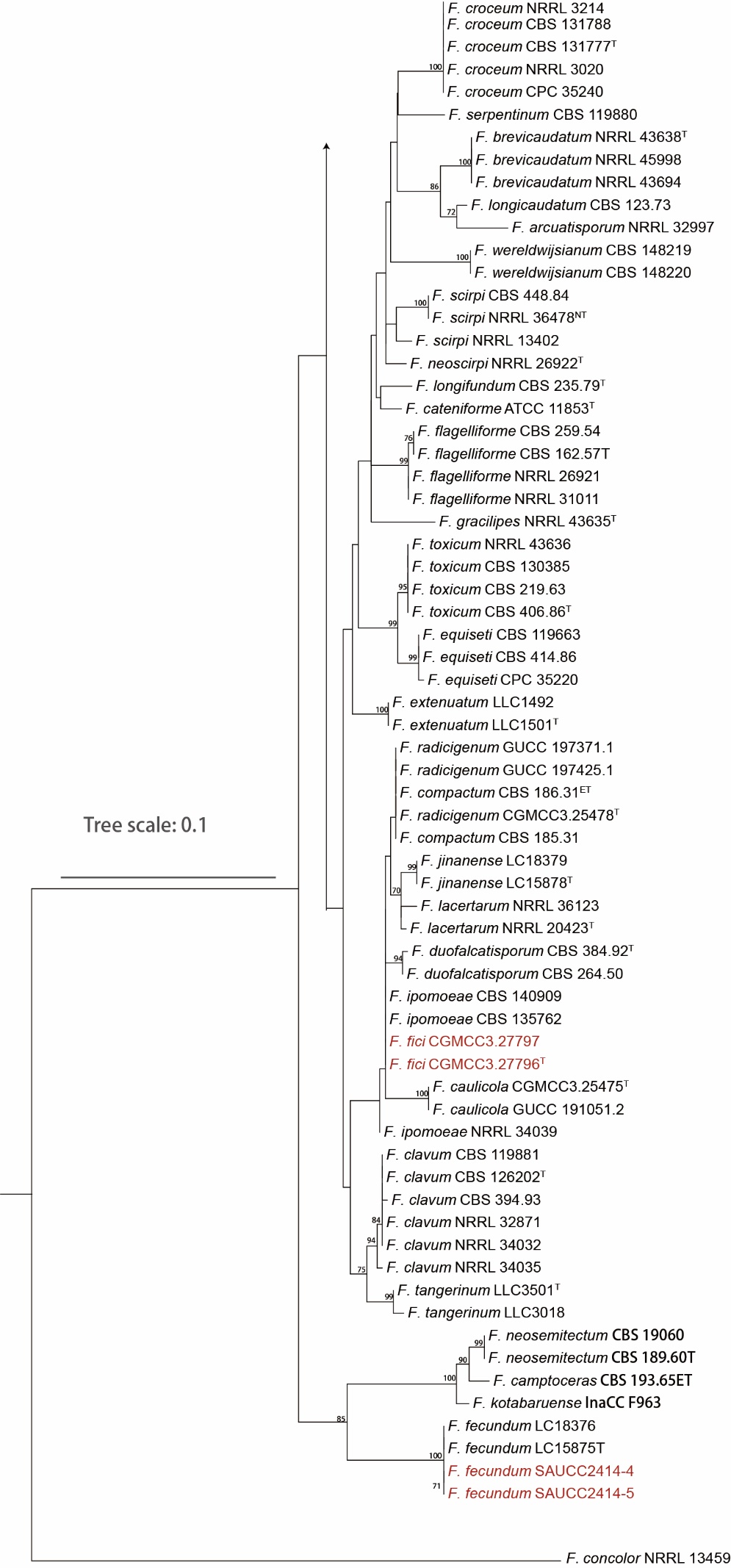


**Supplementary material 2.** Phylogeny of the *Fusarium incarnatum-equiseti* species complex (FIESC) inferred based on the *cal* (a), *rpb2* (b), and *tef1* (c) loci, respectively. *Fusarium concolor* (NRRL 13459) was used as the outgroup. Strains sequenced in this study were indicated in red. The RAxML Bootstrap support values (MLBS ≥ 70 %) were displayed at the nodes. Ex-type, ex-epitype and ex-neotype strains were indicated with T, ET, and NT, respectively.

(c)
