# Supplementary material for: ﻿Morphological and phylogenetic analyses reveal new species and records of Fusarium (Nectriaceae, Hypocreales) from China
Source: MycoKeys. 2025 Apr 7;116:53–71. doi: 10.3897/mycokeys.116.150363 (PMC11997610; doi:10.3897/mycokeys.116.150363)
Supplement: Supplementary material 3 — GenBank accession numbers of the taxa used in phylogenetic reconstruction (Suppl. material 4) [file mycokeys-116-053-s003.docx]

**Supplementary material 3.** GenBank accession numbers of the taxa used in phylogenetic reconstruction (Suppl. material 4)

| Species | Culture accession | Host/substrate | GenBank accession numbers | | |
| --- | --- | --- | --- | --- | --- |
|  |  |  | ***cal*** | ***rpb2*** | ***tef1*** |
| \| *F. weifangense* \| \| --- \| \| *F. weifangense* \| \| *F. weifangense* \| \| *F. weifangense* \| \| *F. weifangense* \| \| *F. weifangense* \| \| *F. weifangense* \| \| *F. weifangense* \| \| *F. weifangense* \| \| *F. citri* \| \| *F. citri* \| \| *F. weifangense* \| \| *F. weifangense* \| \| ***F. weifangense*** \| \| ***F. weifangense*** \| \| *F. concolor* \| | \| CGMCC 3.24285 T = LC18333 ^T^ \| \| --- \| \| LC18311 = HSL1713 \| \| LC18317 = HSL1741 \| \| LC18243 = HSL102 \| \| LC7922 \| \| CBS 130905 \| \| CBS 621.87 \| \| CPC 35143 = CCF 1881 \| \| LMSF-Id01 \| \| LC6896 = CGMCC3.19467 ^T^ \| \| NRRL 52765 = ARSEF 2304 \| \| GUCC 191050.1 = CGMCC 3.25474 \| \| GUCC 191050.2 \| \| **SAUCC 5208C-2** = **CGMCC 3.27939** \| \| **SAUCC 5208C-3** \| \| NRRL 13459 \| | \| Wheat \| \| --- \| \| Wheat \| \| Wheat \| \| Wheat \| \| *Capsicum* sp. \| \| *Triticum* sp. \| \| *Medicago sativa* \| \| *Lactuca sativa* \| \| *Chenopodium quinoa* \| \| *Citrus reticulata* \| \| *Heteropsylla cubana* \| \| *Rosaceae roxburghii* \| \| *Rosaceae roxburghii* \| \| ***Prunus salicina*** \| \| ***Prunus salicina*** \| \| — \| | \| OQ125276 \| \| --- \| \| OQ125274 \| \| OQ125275 \| \| OQ125273 \| \| MK289687 \| \| MN170320 \| \| MN170318 \| \| MN170321 \| \| MW971524 \| \| MK289668 \| \| — \| \| OR043731 \| \| OR043732 \| \| **PQ309117** \| \| **PQ309118** \| \| GQ505585 \| | \| OQ125515 \| \| --- \| \| OQ125514 \| \| OQ125516 \| \| OQ125513 \| \| MK289788 \| \| MN170387 \| \| MN170385 \| \| MN170388 \| \| MW971529 \| \| MK289771 \| \| JF741165 \| \| OR043826 \| \| OR043827 \| \| **PQ309125** \| \| **PQ309126** \| \| GQ505852 \| | \| OQ125107 \| \| --- \| \| OQ125108 \| \| OQ125109 \| \| OQ125106 \| \| MK289634 \| \| MN170454 \| \| MN170452 \| \| MN170455 \| \| MW971534 \| \| MK289617 \| \| JF740839 \| \| OR043881 \| \| OR043882 \| \| **PQ309127** \| \| **PQ309128** \| \| GQ505674 \| |

Ex-type, ex-epitype and ex-neotype strains were indicated in bold with T, ET, and NT, respectively, strains in this study are marked in bold.
