# Supplementary material for: ﻿Morphological and phylogenetic analyses reveal new species and records of Fusarium (Nectriaceae, Hypocreales) from China
Source: MycoKeys. 2025 Apr 7;116:53–71. doi: 10.3897/mycokeys.116.150363 (PMC11997610; doi:10.3897/mycokeys.116.150363)
Supplement: Supplementary material 4 — Phylogeny inferred based on the combined cal-rpb2-tef1 sequence dataset with Fusariumconcolor (NRRL 13459) as the outgroup [file mycokeys-116-053-s004.docx]

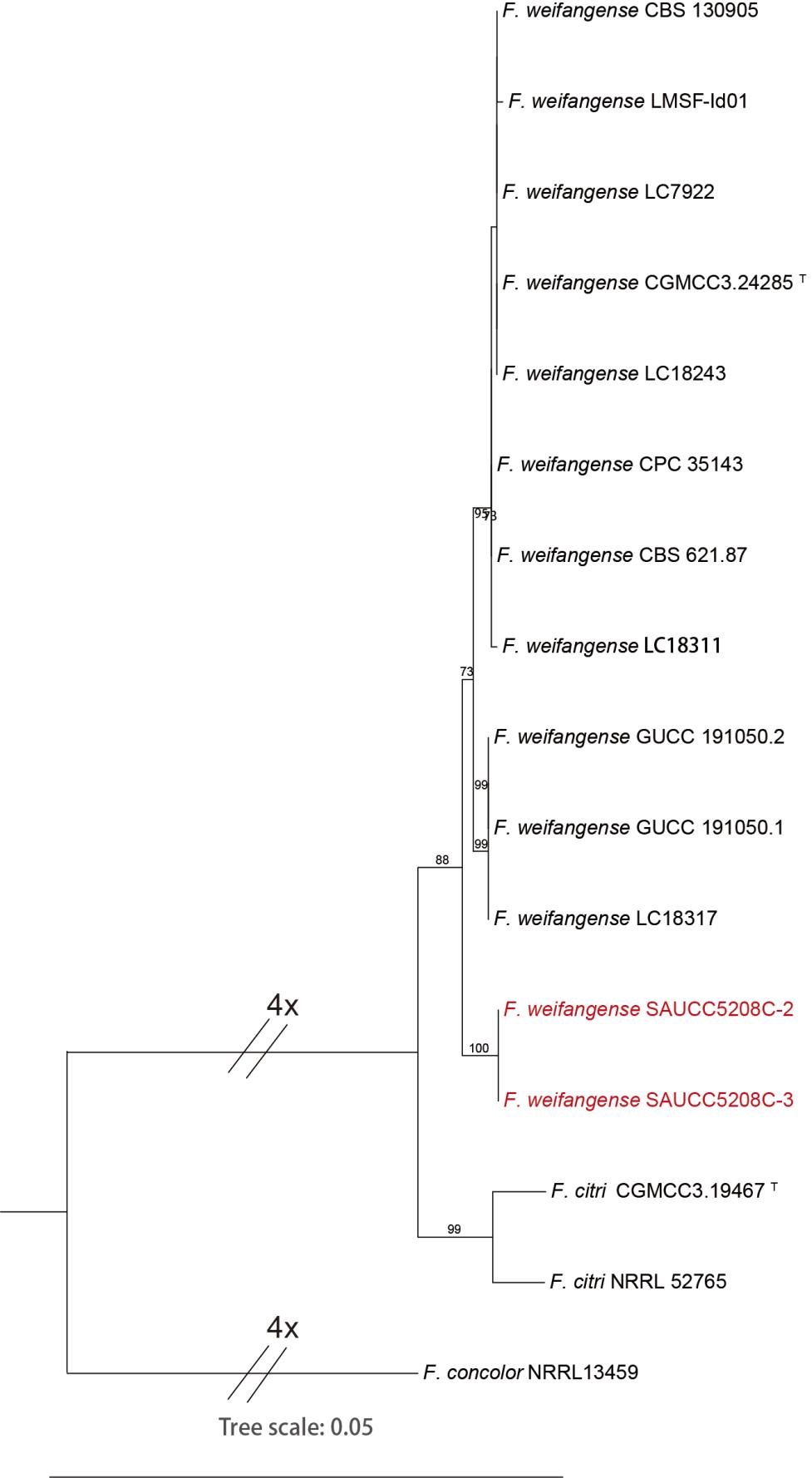


**Supplementary material 4.** Phylogeny inferred based on the combined *cal*-*rpb2*-*tef1* sequence dataset with *Fusarium concolor* (NRRL 13459) as the outgroup. The RAxML Bootstrap support values (MLBS ≥ 70%) were displayed at the nodes. Ex-type, ex-epitype and ex-neotype strains were indicated by T, ET and NT, respectively. Strains isolated in this study were indicated in red.
